# Dynamic brain PET/MR using TOF reconstruction

**DOI:** 10.1186/2197-7364-2-S1-A60

**Published:** 2015-05-18

**Authors:** Mohammad Mehdi Khalighi, Gaspar Delso, Michel Tohme, Andrei Iagaru, Greg Zaharchuk

**Affiliations:** GE Healthcare, USA; UniversitätsSpital Zürich, Switzerland; Stanford University, USA

In a functional PET/MR study, it is difficult to get good temporal resolution of activity distribution from PET images because of the need to image for a certain length of time to get sufficient count statistics (image SNR). Time-of-flight (TOF) reconstruction can be used to increase PET images SNR and therefore increase the temporal resolution. Five patients were injected with 410±80 MBq of FDG and scanned 140±30 minutes post-injection on a simultaneous TOF-enabled PET/MR scanner. PET images were reconstructed with and without TOF. TOF reconstruction shows faster convergence while it achieves a temporal SNR improvement of 5-45% (25±5%) compared to non-TOF reconstruction. With this additional SNR gain, frame durations as short as 30s are possible while preserving reasonable image quality. This in turn effectively increases the temporal resolution of dynamic brain studies using simultaneous PET/MR imaging.

